# Supplementary material for: On the Operational Utility of Measures of Multichannel EEGs
Source: Entropy (Basel). 2021 Oct 30;23(11):1434. doi: 10.3390/e23111434 (PMC8617715; doi:10.3390/e23111434)
Supplement: Supplementary file 1 [file entropy-23-01434-s001.zip › Supplementary files.pdf]

Supplement Darmon et al. On the operational utility of measures of multichannel EEGs

Mathematical description of Integrated Synergy
Table S1: Mean and Standard Deviation: Signals Containing Alpha Band
Table S2: Mean and Standard Deviation: Signals with Alpha Band Removed
Table S3: Effect Size: Eyes Open v. Eyes Closed, Robust Estimator, Signals Containing Alpha Band
Table S4: Effect Size: Eyes Open v. Eyes Closed, Robust Estimator, Signals With Alpha Band Removed
Figure S1: Effect Size: Eyes Open v. Eyes Closed, Robust Estimator: (A). Alpha band present, (B). Alpha band removed
Figure S2: Difference scores for each measure (eyes open - eyes closed)
Table S5: Kendall's tau correlation: Eyes Closed, Alpha Band Present
Table S6: Kendall's tau correlation: Eyes Closed, Alpha Removed
Table S7: Kendall's tau correlation: Eyes Open, Alpha Band Present
Table S8: Kendall's tau correlation: Eyes Open, Alpha Band Removed
Figure S3: Within-subject association alpha component present
Figure S4: Within-subject association alpha component removed

Mathematical Description Integrated Synergy

Let $\mathcal{P}$ be a particular partition of the state vector $\mathbf{V} = (V_1, \ldots, V_d)$ into r state vectors $(\mathbf{M}^{(1)}, \mathbf{M}^{(2)}, \ldots, \mathbf{M}^{(r)})$. Then the integrated synergy ψ_1 is defined as the decrease in the mutual information between the most-recent past and present of the system when using a partition of the most-recent past that maximizes a certain notion of non-redundant information between the partition elements and the present,

$$\psi_1 = I\left[\mathbf{V}_{t-1} \wedge \mathbf{V}_t\right] - \max_{\mathcal{P}} I_{\cup}\left[\left(\mathbf{M}_{t-1}^{(1)}, \ldots, \mathbf{M}_{t-1}^{(r)}\right) \wedge \mathbf{V}_t\right], \tag{1}$$

where $I_{\cup}\left[\left(\mathbf{M}_{t-1}^{(1)}, \ldots, \mathbf{M}_{t-1}^{(r)}\right) \wedge \mathbf{V}_t\right]$ is the intersection information between a particular partition of $\mathbf{V}_{t-1}$ and $\mathbf{V}_t$ (Mediano, et al.). The intersection information is defined via an inclusion-exclusion principle as

$$I_{\cup}\left[\left(\mathbf{M}_{t-1}^{(1)}, \ldots, \mathbf{M}_{t-1}^{(r)}\right) \wedge \mathbf{V}_t\right] = \sum_{\mathcal{S} \in \mathcal{P}^{+}(\{\mathbf{M}^{(1)}, \ldots, \mathbf{M}^{(r)}\})} (-1)^{|\mathcal{S}|+1} I_{\cap}\left[\left(\mathbf{S}_{t-1}^{(1)}, \ldots, \mathbf{S}_{t-1}^{(|\mathcal{S}|)}\right) \wedge \mathbf{V}_t\right]. \tag{2}$$

where $\mathcal{P}^{+}(\{\mathbf{M}^{(1)}, \ldots, \mathbf{M}^{(r)}\})$ is the power set of $\{\mathbf{M}^{(1)}, \ldots, \mathbf{M}^{(r)}\}$ excluding the empty set and $I_{\cap}\left[\left(\mathbf{S}_{t-1}^{(1)}, \ldots, \mathbf{S}_{t-1}^{(|\mathcal{S}|)}\right) \wedge \mathbf{V}_t\right]$ is the intersection information, i.e. the redundant information, between a subset $\mathcal{S}$ of the partition and $\mathbf{V}_t$ (Griffith).

For a linear Gaussian VAR, the union information reduces to the maximum over the mutual informations between the present state and each single element of the partition

$$I_{\cup}\left[\left(\mathbf{M}_{t-1}^{(1)}, \ldots, \mathbf{M}_{t-1}^{(r)}\right) \wedge \mathbf{V}_t\right] = \max_{k} I\left[\mathbf{M}_{t-1}^{(k)} \wedge \mathbf{V}_t\right]. \tag{3}$$

(Olbrich, Bertschinger, and Rauh).

As in (Mediano, et al.), we restrict our analysis to equal-sized bipartitions of the state vector. This is done to avoid bias due to differences in integrated information between different-sized partitions.

Order-p Integrated Synergy

Supplement Darmon et al. On the operational utility of measures of multichannel EEGs

The integrated synergy (1) implicitly assumes that the stochastic process governing the state variable is an order-1 Markov process. That is, it assumes that knowledge of the most recent past is sufficient to predict the future of the time series. There is no reason to assume this a priori, especially for time series derived from electrophysiology. In principle, one could allow for the entire past of the time series to be necessary for optimal prediction, i.e. if the process is a linear stochastic process. In this section, we define this infinite-order integrated synergy as well as an order-p compromise.

Let $\mathbf{Z}_{a:b} = (\mathbf{Z}_a, \mathbf{Z}_{a+1}, \ldots, \mathbf{Z}_{b-1})$ be the random vector containing the state vector from time points a to $b-1$. Then the infinite order integrated synergy ψ_∞ is defined as

$$\psi_\infty = I\left[\mathbf{V}_{-\infty:t} \wedge \mathbf{V}_t\right] - \max_{\mathcal{P}} I_\cup \left[\left(\mathbf{M}^{(1)}_{-\infty:t}, \ldots, \mathbf{M}^{(r)}_{-\infty:t}\right) \wedge \mathbf{V}_t\right]. \tag{4}$$

In practice, with finite data available to estimate the time series model, finite orders are necessary. Truncating at sufficiently large lag p into the past gives the order-p integrated synergy ψ_p,

$$\psi_p = I\left[\mathbf{V}_{t-p:t} \wedge \mathbf{V}_t\right] - \max_{\mathcal{P}} I_\cup \left[\left(\mathbf{M}^{(1)}_{t-p:t}, \ldots, \mathbf{M}^{(r)}_{t-p:t}\right) \wedge \mathbf{V}_t\right], \tag{5}$$

where all quantities are defined as in the order-1 case with the inclusion of additional lags.

In general, a vector stochastic process that is VAR(p) overall need not be VAR(p) in any of its components (Lütkepohl), and thus infinite orders may be necessary for the $I_\cup \left[\left(\mathbf{M}^{(1)}_{t-p:t}, \ldots, \mathbf{M}^{(r)}_{t-p:t}\right) \wedge \mathbf{V}_t\right]$ term. While we do not pursue this avenue here, one approach would be to take sufficiently many lags into the past based on the spectral radius of the coefficient matrix from the VAR model.

Estimating Order-1 and Order-p Integrated Synergy

In all cases, we estimate the integrated synergy using a plug-in estimate from a linear Gaussian VAR model fit to the data using ordinary least squares, using (3) or its order-p generalization to compute the union information in (1) or (5). For the order-p integrated synergy, the model order p is chosen using Schwarz's Bayesian Information Criterion assuming a linear Gaussian VAR model (Lütkepohl) with a maximum possible model order of $p_{\max} = 60$. See the Section 4.1 of (Mediano, et al.) for more details on estimating the linear Gaussian VAR and computing its associated information-theoretic properties.

References

Griffith, V. A principled infotheoretic Φ-like measure. arXiv: 1401.0978v3 [cs.IT]

Lütkepohl, H. New Introduction to Multiple Time Series Analysis., **2005**, New York, NY: Springer Science and Business Media.

Mediano, P.A.M., Seth, A.K. and Barrett, A.B. Measuring integrated information: comparison of candidate measures in theory and simulation. *Entropy.* **2019**, 21, 77

Olbrich, E. Bertschinger, N. and Rauh, J. Information decomposition and synergy. *Entropy* **2015**, 17, 3501-3517.

Tononi,G., Sporns,O. and Edelman,G. A measure for brain complexity: Relating functional segregation and integration in the nervous system. *Proceedings National Academy of Science (USA)* **1994**, 91(11), 5033-5037.

Table S1. Mean and Standard Deviation
Signals containing the alpha component

Measure	Eyes Closed Mean±StDev	Eyes Open Mean±StDev
Binary Lempel Ziv Signals mean normalized	174.250 ±32.811	237.667 ±47.483
Binary Lempel Ziv Signals normalized by mean and by standard deviation	188.250 ±34.489	239.500 ±38.339
C_N Tononi, et al. 1994 Equation 4	5.5095 ±0.659	4.863 ±0.657
ψ $p = 1$ Mediano, et al. 2019 Equation 23	6.415 ±0.942	5.559 ±1.017
ψ p via BIC Mediano, et al. Equation 23	0.2535 ±0.0843	0.4548 ±0.1569

Table S2. Mean and Standard Deviation
Signals with the alpha component removed

Measure	Eyes Closed Mean±StDev	Eyes Open Mean±StDev
Binary Lempel Ziv Signals mean normalized	230.667 ±47.266	273.417 ±46.800
Binary Lempel Ziv Signals normalized by mean and by standard deviation	227.250 ±38.248	266.917 ±34.935
C_N Tononi, et al. 1994 Equation 4	4.8328 ±0.760	4.623 ±0.646
ψ $p = 1$ Mediano, et al. 2019 Equation 23	5.687 ±0.802	5.012 ±0.847
ψ p via BIC Mediano, et al. Equation 23	0.3717 ±0.1922	0.4396 ±0.1591

Table S3. Effect Size: Eyes Open - Eyes Closed
Signals containing the alpha component, robust estimator

Measure	Estimated Effect Size	95% Confidence Interval
Binary Lempel Ziv<br>Signals mean normalized	1.37	(1.025, 2.665)
Binary Lempel Ziv<br>Signals normalized by mean and by standard deviation	1.14	(0.881, 2.280)
C_N<br>Tononi, et al. 1994<br>Equation 4	-1.48	(-4.121, -0.829
ψ $p = 1$<br>Mediano, et al. 2019<br>Equation 23	-0.75	(-1.537, -0.501
ψ p via BIC<br>Mediano, et al. 2019<br>Equation 23	0.79	(0.233, 2.003)

Table S4. Effect Size: Eyes Open - Eyes Closed
Signals with the alpha component removed, robust estimator

Measure	Estimated Effect Size	95% Confidence Interval
Binary Lempel Ziv<br>Signals mean normalized	0.89	(0.269, 2.030
Binary Lempel Ziv<br>Signals normalized by mean and by standard deviation	1.63	(0.412, 6.783)
C_N<br>Tononi, et al. 1994<br>Equation 4	-0.28	(-1.035, 1.401)
ψ $p = 1$<br>Mediano, et al. 2019<br>Equation 23	-1.16	(-3.106, -0.344
ψ p via BIC<br>Mediano, et al. 2019<br>Equation 23	-0.03	(-1.015, 2.241)

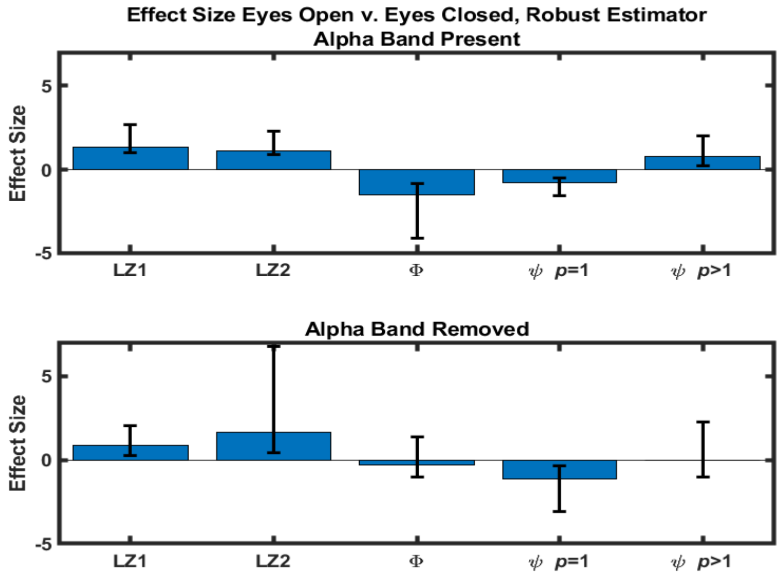

Figure S1. Eyes open versus eyes closed effect size as quantified by the robust estimator for five measures. Confidence intervals were determined with a bias-correct and adjusted bootstrap.
(A). Effect sizes calculated with signals that contain the alpha component.
(B). Effect sizes calculated after the alpha component had been removed with an 8 Hz to 13 Hz stopband filter.

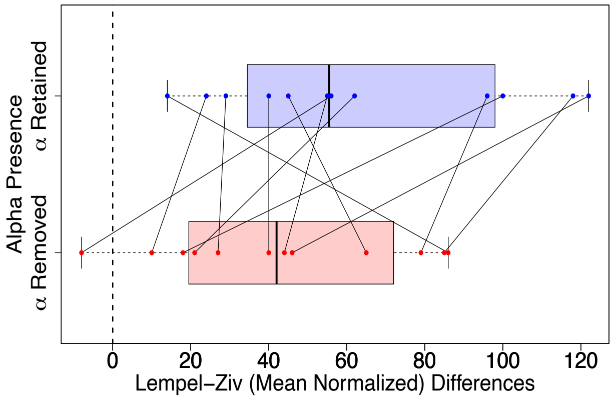

(a)

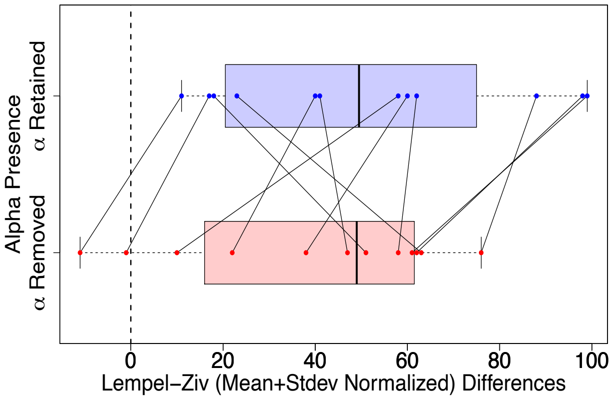

(b)

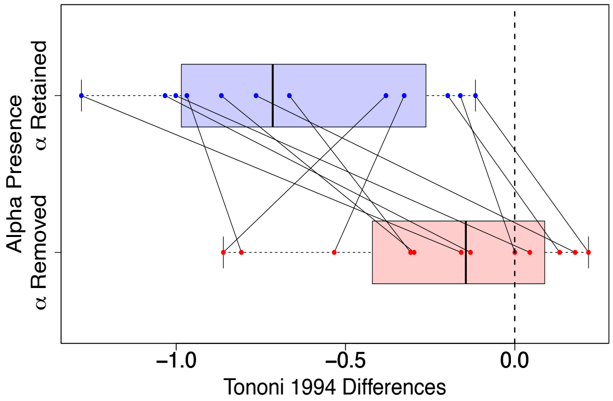

(c)

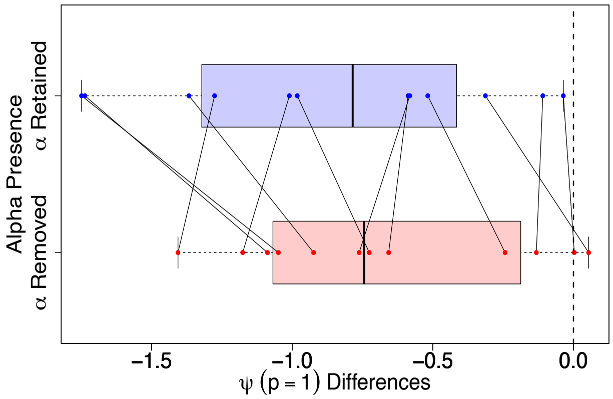

(d)

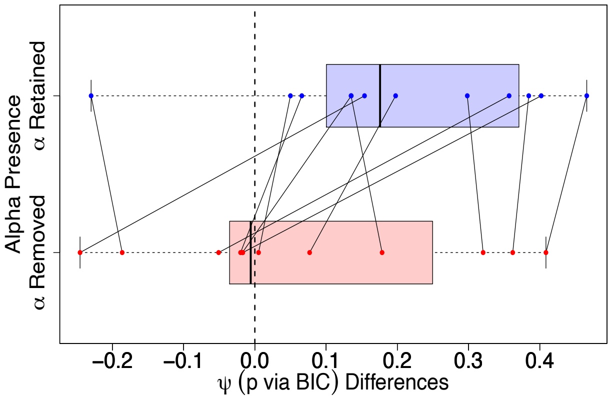

(e)

Figure S2. The difference scores for each measure across the subjects with (red) and without (blue) the alpha component removed. Solid lines match subjects between alpha retained and alpha removed conditions. The dash vertical line indicates no difference between the eyes-open and eyes-closed conditions.

Table S5. Kendall's tau correlation
Eyes Closed, Alpha Component Present

	LZ1	LZ2	Φ	ψ $p = 1$	ψ p via BIC
LZ1	1.0000	0.7273	-0.2424	-0.0909	0.2424
LZ2		1.0000	-0.3333	-0.3636	0.2121
Φ			1.0000	0.2424	-0.1515
ψ $p = 1$				1.0000	-0.5455
ψ p via BIC					1.0000

Table S6. Kendall's tau correlation
Eyes Closed, Alpha Component Removed

	LZ1	LZ2	Φ	ψ $p = 1$	ψ p via BIC
LZ1	1.0000	0.6364	-0.1818	0.0909	0.1818
LZ2		1.0000	-0.3030	0.0303	0.1212
Φ			1.0000	-0.1212	0.0303
ψ $p = 1$				1.0000	-0.4242
ψ p via BIC					1.0000

Table S7. Kendall's tau correlation
Eyes Open, Alpha Component Present

	LZ1	LZ2	Φ	ψ $p = 1$	ψ p via BIC
LZ1	1.0000	0.9313	-0.0763	-0.4428	0.1679
LZ2		1.0000	-0.0606	-0.3939	0.0909
Φ			1.0000	0.0000	-0.2424
ψ $p = 1$				1.0000	-0.0909
ψ p via BIC					1.0000

Table S8. Kendall's tau correlation
Eyes Open, Alpha Component Removed

	LZ1	LZ2	Φ	ψ $p = 1$	ψ p via BIC
LZ1	1.0000	0.5198	-0.0153	-0.2595	-0.1069
LZ2		1.0000	-0.0313	-0.2189	-0.0938
Φ			1.0000	-0.2121	-0.0606
ψ $p = 1$				1.0000	-0.1212
ψ p via BIC					1.0000

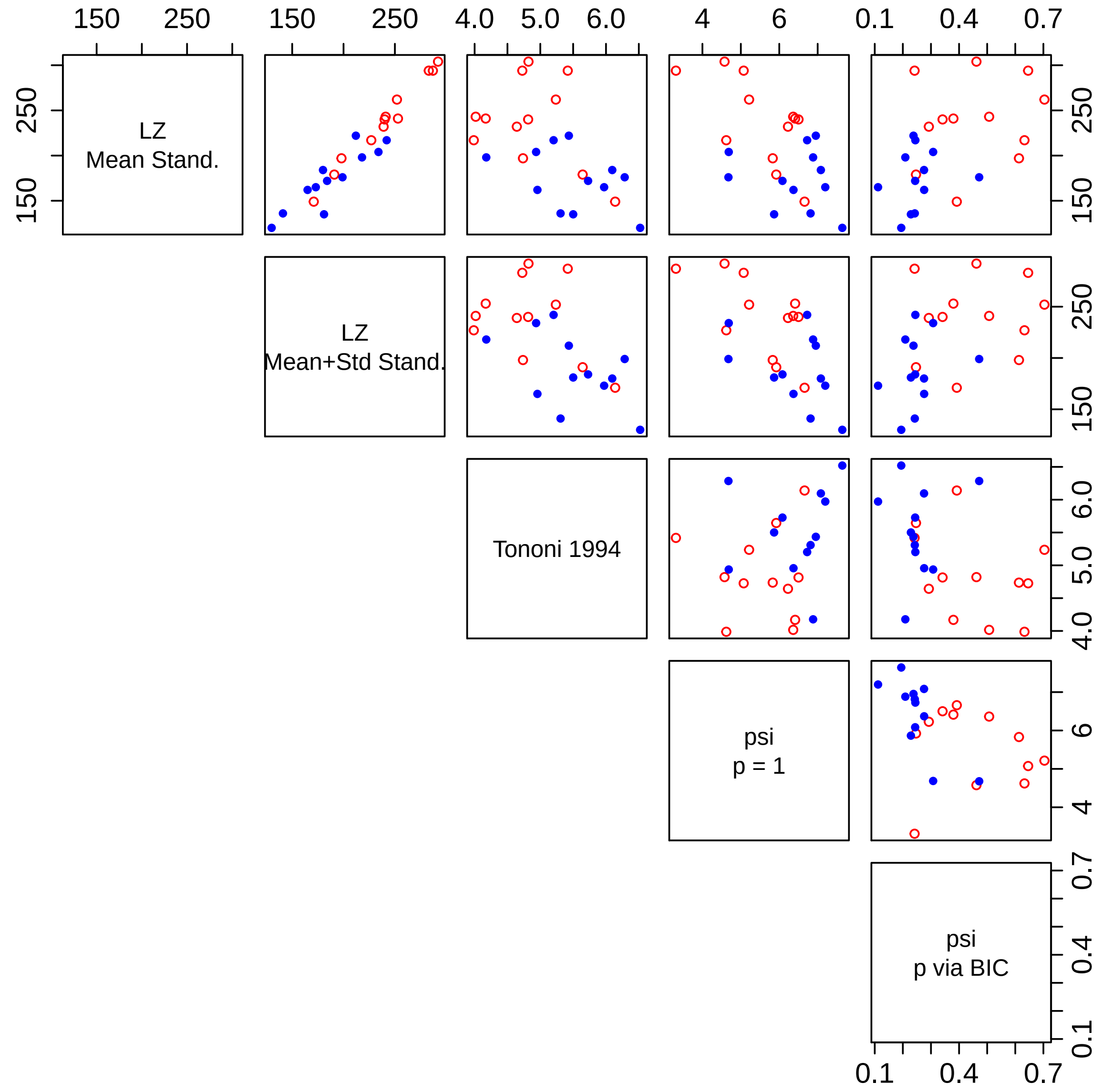

Figure S3. Within-subject assocations between the measures in the eyes-open (open red points) and eyes-closed conditions (closed blue points) from signals containing the alpha component.

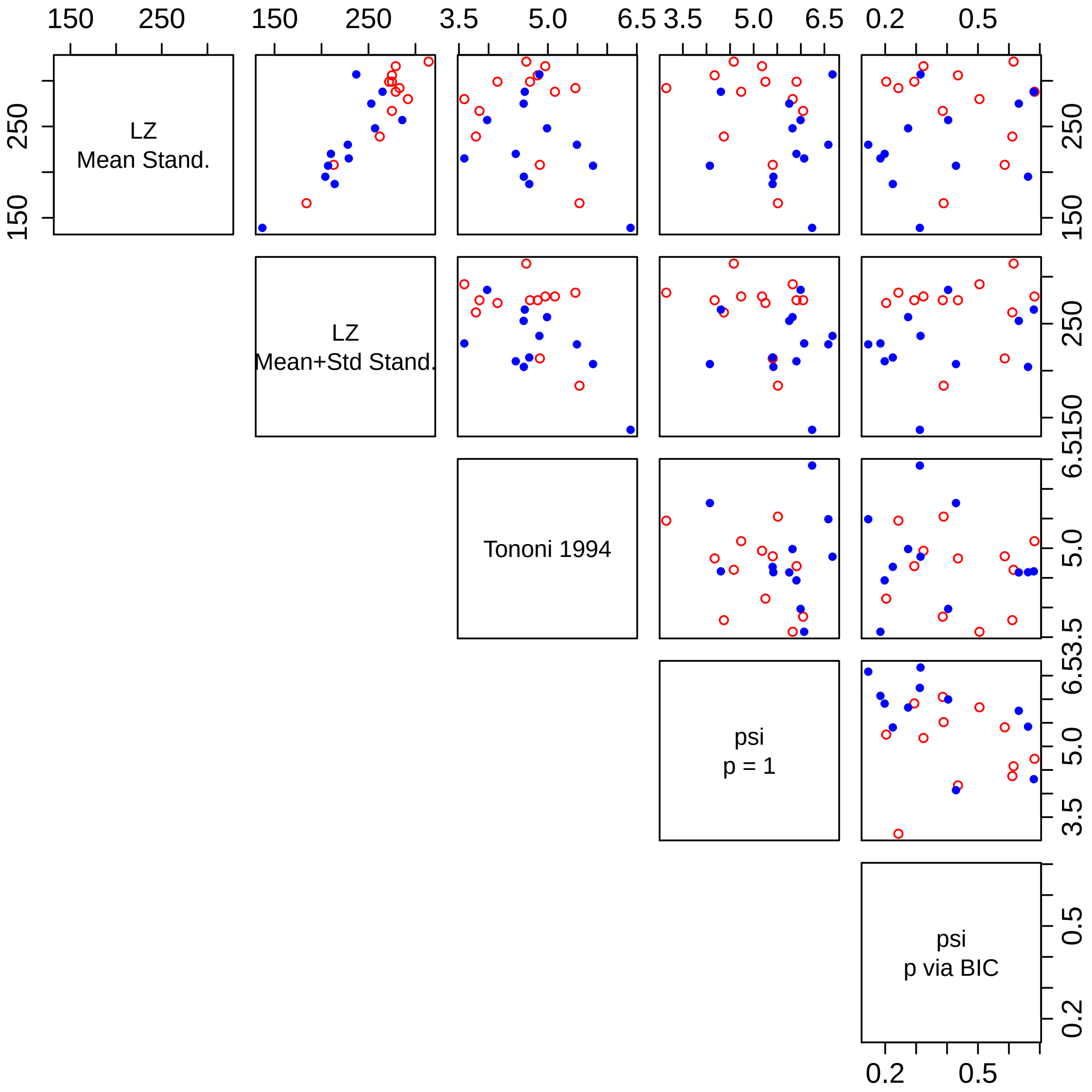

Figure S4. Within-subject assocations between the measures in the eyes-open (open red points) and eyes-closed conditions (closed blue points). The alpha component has been removed.
